# Body-machine interface for control of a screen cursor for a child with congenital absence of upper and lower limbs: a case report

**DOI:** 10.1186/s12984-016-0139-4

**Published:** 2016-03-24

**Authors:** Mei-Hua Lee, Rajiv Ranganathan, Florian A. Kagerer, Ranjan Mukherjee

**Affiliations:** Department of Kinesiology, Michigan State University, 308 W Circle Dr Rm 201, East Lansing, MI 48824 USA; Department of Mechanical Engineering, Michigan State University, East Lansing, MI USA

## Abstract

**Background:**

There has been a recent interest in the development of body-machine interfaces which allow individuals with motor impairments to control assistive devices using body movements.

**Methods:**

In this case study, we report findings in the context of the development of such an interface for a 10-year old child with congenital absence of upper and lower limbs. The interface consisted of 4 wireless inertial measurement units (IMUs), which we used to map movements of the upper body to the position of a cursor on a screen. We examined the learning of a task in which the child had to move the cursor to specified targets on the screen as quickly as possible. In addition, we also determined the robustness of the interface by evaluating the child’s performance in two different body postures.

**Results:**

We found that the child was not only able to learn the task rapidly, but also showed superior performance when compared to typically developing children in the same age range. Moreover, task performance was comparable for the two different body postures, suggesting that the child was able to control the device in different postures without the need for interface recalibration.

**Conclusions:**

These results clearly establish the viability and robustness of the proposed non-invasive body-machine interface for pediatric populations with severe motor limitations.

## Background

Assistive devices facilitate interaction with both the physical world (for example using prosthetic limbs, powered wheelchairs), as well as the virtual world (for example using a pointing device to type or browse the web), and play a critical role in maintaining independence in activities of daily living (ADLs) for people with movement impairments. Although assistive devices typically have their own control interface (e.g., a joystick for a wheelchair, switches for a prosthetic arm), there is a need for designing a general-purpose human-machine interface that can ‘plug into’ a variety of devices, especially for people with severe impairments who may not be able to use device-specific controllers. In this context, there has been tremendous progress in the area of brain-machine interfaces where signals are recorded from the brain (either invasively or non-invasively) in order to control external devices [10, 18, 29]. However, it is important to recognize that there are significant disadvantages with both invasive and non-invasive brain-machine interfaces - invasive brain-machine interfaces involve surgical risks and system durability issues [24], whereas non-invasive interfaces involve low signal-to-noise ratios and susceptibility to signal artifacts [18]. As a result, the main focus of these interfaces has been for people with an almost complete absence of movement.

In this context, it is worth noting that movements of the body are still possible in a significant proportion of people with movement impairments. Body-machine interfaces [4, 21] attempt to exploit these residual movements by using signals from body movements (instead of neural signals) to control external devices. Body-machine interfaces for people with amputations or paralysis have tapped into a wide range of signals including electromyography [17], electro-oculography [1], and tongue movements [16]. Here, we focus on body-machine interfaces that tap into movement kinematics of the musculoskeletal system (e.g., data measured through motion capture, inertial measurement units, accelerometers etc.). Previous work on body-machine interfaces has shown that they can be used for people with limited mobility such as for individuals with spinal cord injury [5], and that, on a neural level, prolonged use of these interfaces can result in reorganization of white matter tracts in the brain [28].

One critical question is the applicability of body-machine interfaces in children, as there are both theoretical and practical issues that deserve special attention in this population. For example, when using simple computer interfaces like a mouse, younger children show lower performance than adults (curved trajectories and slower movement times), and specifically have difficulty stopping near the target [12]. In addition, from a cognitive standpoint, there are developmental constraints related to memory and information processing that may influence the learning of such complex interfaces [2, 8, 26]. Finally, from a practical standpoint, barriers to the use of assistive devices in children include (i) complexity of the device - which often necessitates significant training for the caregiver and school personnel [7], and (ii) physical appearance of the interface – i.e. children may avoid devices that are easily noticeable (such as those with wires or bulky attachments) in order to avoid being seen as different from their peers [14].

In the current study, we examined the use of body-machine interface based on a wireless inertial-measurement system in a child with congenital absence of upper and lower limbs. We examined how the child learned a cursor-control task using the body-machine interface. In addition, since the IMUs are sensitive to orientation in space, we also evaluated the robustness of the interface over multiple days and by examining his performance in two different body postures. Our results show that the system is both easy to learn and robust, making it feasible for the use of control of assistive devices in this population.

## Methods

### Participant

The child (‘P1’) was a 10-year old boy with congenital absence of both upper and lower limbs. His cognitive status was normal, and there was no history of any neurological or orthopedic conditions. His only prior experience with a device interface was the control of a powered wheelchair with a joystick, which he controlled with his right shoulder. P1 and his parents provided informed consent; the procedures in the study were approved by the Michigan State University IRB.

### Experimental setup and protocol

#### Apparatus

**Fig. 1:**
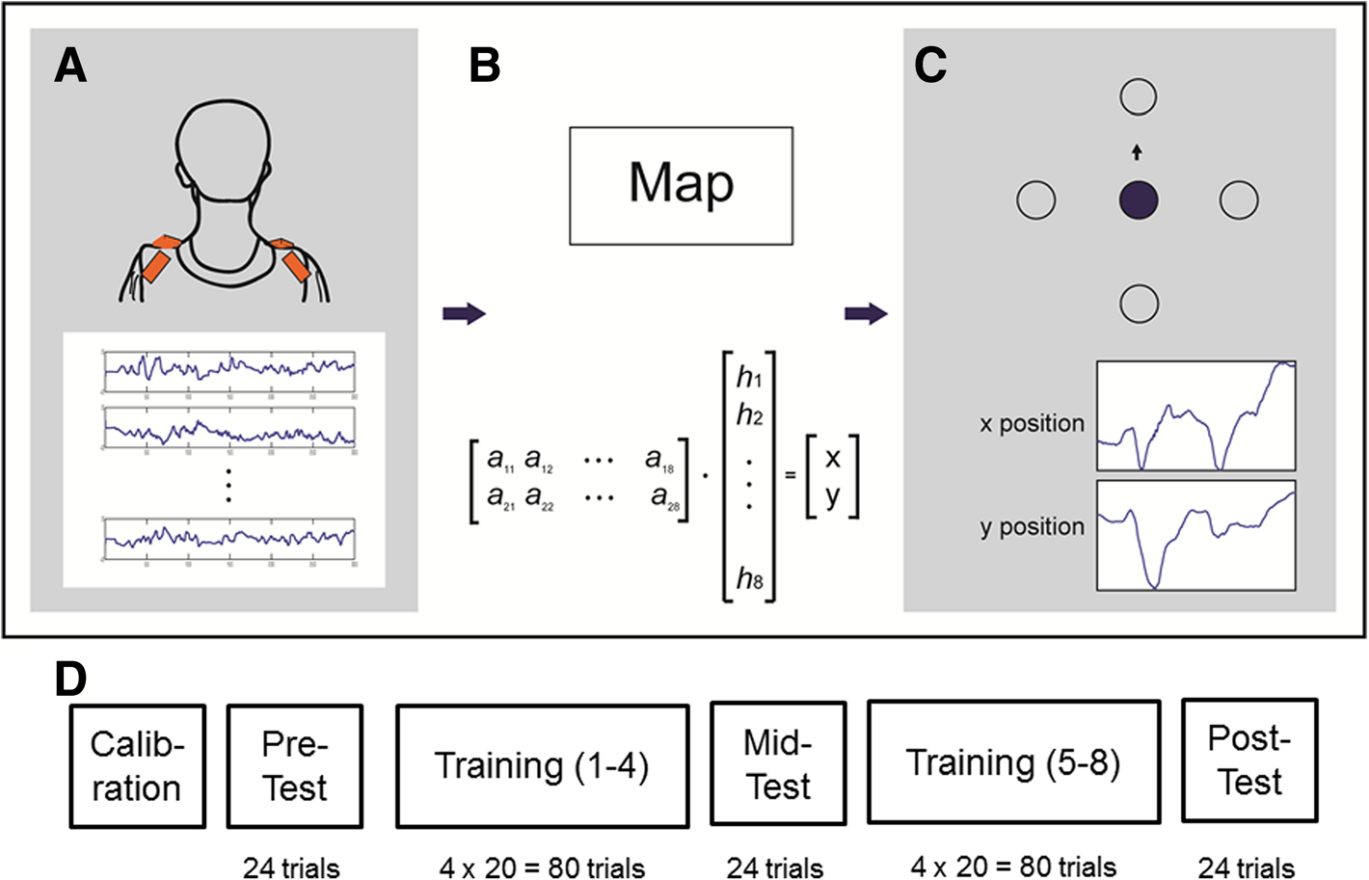
Schematic of the body-machine interface (**a**) 4 IMUs are attached to the upper body that measure movement of the upper body. **b** These signals are transformed by a map A into the x-y position of a cursor. **c** The position of the cursor is displayed on a screen and the participant is asked to move the cursor to different targets. **d** Schematic of the experimental protocol in one session – after an initial calibration phase, participants are asked to perform a series of training and test blocks to evaluate motor learning. The calibration block was only performed on the first experimental session, whereas the remaining blocks were performed in each experimental session

P1 was seated in front of a 23” computer monitor, at a distance of approximately 70 cm. Four wireless IMUs (3-space, YEI Technology, Portsmouth, OH, USA) were attached to his upper body using a customized vest and velcro loops The IMUs were attached to the front and back of the trunk just proximal to the left and right acromioclavicular joint (Fig. 1a). The sampling rate of the IMUs was set at 50 Hz. These signals were then transformed by a linear map (Fig. 1b) into x-y coordinates of a screen cursor (Fig. 1c). The 4 IMUs were used to capture a combination of scapular retraction, protraction, elevation and depression [9], and the number of IMUs used represented a balance between capturing a rich set of movements, and being convenient enough for home use. We used a custom program in MATLAB/Simulink (Mathworks, Natick, MA) for acquiring the IMU signals and then transforming them to cursor position.

### Calibration

In order to determine the ‘map’ that would be used to transform the IMU signals to cursor position, we initially had P1 perform a “body dance” for 60 sec [23] in which he was asked to perform exploratory movements with his upper body while maintaining a comfortable range of motion. Each IMU provided 3 signals (roll, pitch, and yaw angles) but for the purposes of the interface we used only 2 of them (roll and pitch). The yaw angle was not used as it depends on the magnetic field and is sensitive to the presence of metals in the environment. No filtering or pre-processing was applied to the IMU signals. We then used principal components analysis (PCA) to extract the first two principal components from the calibration data. The two principal component vectors were of the form: v_1_ = a_11_h_1_ + a_12_ h_2_ + .. + a_18_ h_8_, and v_2_ = a_21_ h_1_ + a_22_ h_2_ + … + a_28_ h_8_ , where h_1_, h_2_,…h_8_ are the signals (roll and pitch angles) of the 4 IMU sensors . The map A was constructed using the coefficients of the two principal component vectors as follows:

$$ A=\left[\frac{a_{11}\kern1em {a}_{12}\cdots \kern1.00em {a}_{18}}{a_{21\kern0.5em }\kern0.5em {a}_{22}\cdots \kern0.5em {a}_{28}}\right] $$

Finally, the two eigen values corresponding to the two principal components, λ_1_ and λ_2 ,_ were used as gain factors to make the task difficulty equivalent along both directions (since principal component 1 will have a higher eigen value than principal component 2). The first row of coefficients in A was scaled by 1/√λ_1_ and the second row by 1/√λ_2_.

From this calibration, the cursor position (p) at any instant was determined from the IMU signal vector (h) by a simple matrix multiplication: *p* = A *h* + *p*_0_, where *p*, *p*_0_ are 2x1 vectors of cursor coordinates (*p*_0_ served as an offset term), A is a 2x8 matrix that defined the map, and *h* is a 8x1 vector of IMU measurements. *p*_0_ was an offset term calculated so that the mean posture during the body dance (which was close to the resting posture) corresponded to the cursor being in the middle of the screen.

### Reaching

After the calibration, P1 was instructed to move his upper body to control the cursor on the computer monitor. In this center-out reaching task, participants reached from a ‘home’ position (in the center of the screen) to one of either four or eight peripheral targets presented, and then returned to the home position (target diameter: 80 pixels, 2.2 cm). Targets were located at a distance of 409 pixels (11.5 cm) from the home position on the screen and were presented in a random sequence. To start each trial, the cursor had to be kept inside the home target for 500 ms, after which a target appeared. Once the target was reached, P1 was also instructed to keep the cursor inside of the target for 500 ms before the home target appeared again. P1 was instructed to move the cursor to the target as fast and accurate (i.e. as close to the center of the target) as possible. A scoring system was also used to encourage fast and accurate task performance. The schedule of the experiment is shown in Fig. 1d. Each trial continued until the participant reached the target, and our primary measure of task performance was the time taken to reach the target.

### Evaluating robustness

Since IMUs are sensitive to orientation in space, we examined the robustness of learning to changes in trunk orientation. Using a motion capture system (Motion Analysis, Santa Rosa, CA) we established that the change in anterior-posterior trunk orientation (when he was in a neutral position) between the two postures was ~8°. Critically, when we changed the body posture, our participant still retained the previously established map that was used when he was seated in the wheelchair (i.e., we did not recalibrate the interface by creating a new map to account for the new posture).

### Practice schedule

In total, there were 4 experimental sessions (each session was held on a different day) that were all separated by approximately 1 week. Sessions 1 and 2 were conducted with P1 seated in his wheelchair, whereas sessions 3 and 4 were conducted with P1 seated in a bucket prosthesis. Each session consisted of the same structure (Fig. 1d) – a pre-test (24 trials), 4 training blocks (4 x 20 = 80 trials), a mid-test (24 trials), 4 more training blocks (4 x 20 = 80 trials), and a post-test (24 trials). The only difference between training and test blocks was the number of targets: in the training blocks, P1 reached for 4 peripheral targets in the cardinal directions. In the test blocks, participants reached to 8 peripheral targets (45 degrees apart) so that we could also examine if performance generalized to untrained targets. Each experimental session lasted for about one hour.

### Control data for comparison

To provide a reference for comparison, we have also included the data from typically developing individuals – two groups of children (aged 9, *n* = 13; aged 12, *n* = 12), and college-aged adults (18-25 years, *n* = 20) for the same task (data from [19]). These control participants performed the same task as P1 in Day 1, but did not perform any of the other sessions (including the change in posture).

### Data analysis

The data were analyzed using the following two metrics:

### Movement time

Movement time was computed as the time taken between the instant when the cursor left the start target to the instant when the cursor first entered the target and stayed in that target for the next 500 ms (note that this was not included as part of the movement time).

### Normalized path length

In order to measure the efficiency of the path taken, we computed the normalized path length by dividing the total distance traveled by the cursor when moving between the targets by the total displacement (i.e. the straight line distance) between the two targets. A perfectly straight path (without any overshoots or reversals) would result in a normalized path length value of 1. The criteria used to determine the start and end of the movement were the same as those used to compute the movement time.

### Statistical analysis

To examine if improvements were statistically significant in P1, we used the non-parametric Mann-Whitney U test to compare the movement time and the path length between: (i) pre-test and post-test on Day 1 (to examine whether there was learning), (ii) pre-test on Day 1 and pre-test on Day 2 (to examine retention after 1 week), and (iii) mid-test on Day 1 and Day 3 (to examine robustness of interface to different postures). We used the mid-test instead of the pre-test because there was missing data on the pre-test of Day 3. The significance level was set at *p* < .05. Also, since the focus of this study was on P1, we did not use any of the data from typically developing individuals for statistical analysis.

## Results

### Movement time

**Fig. 2:**
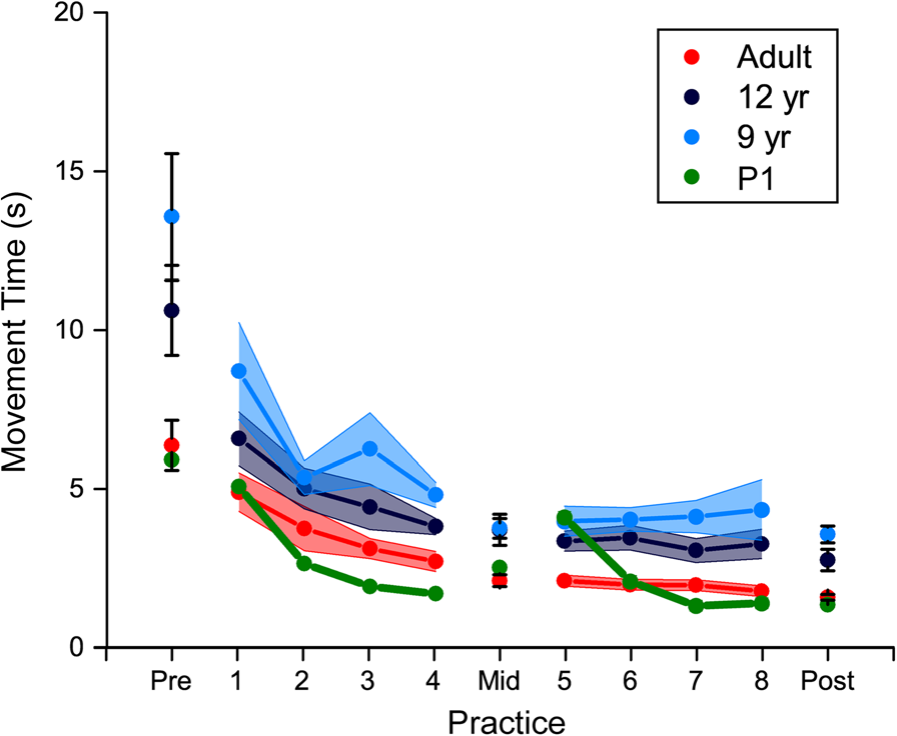
Mean movement time of P1 relative to a sample of typically developing children (9- and 12-year olds) and college-aged adults in the first experimental session (Day 1). Error bars represent 1 standard error (between-participant)

**Fig. 3:**
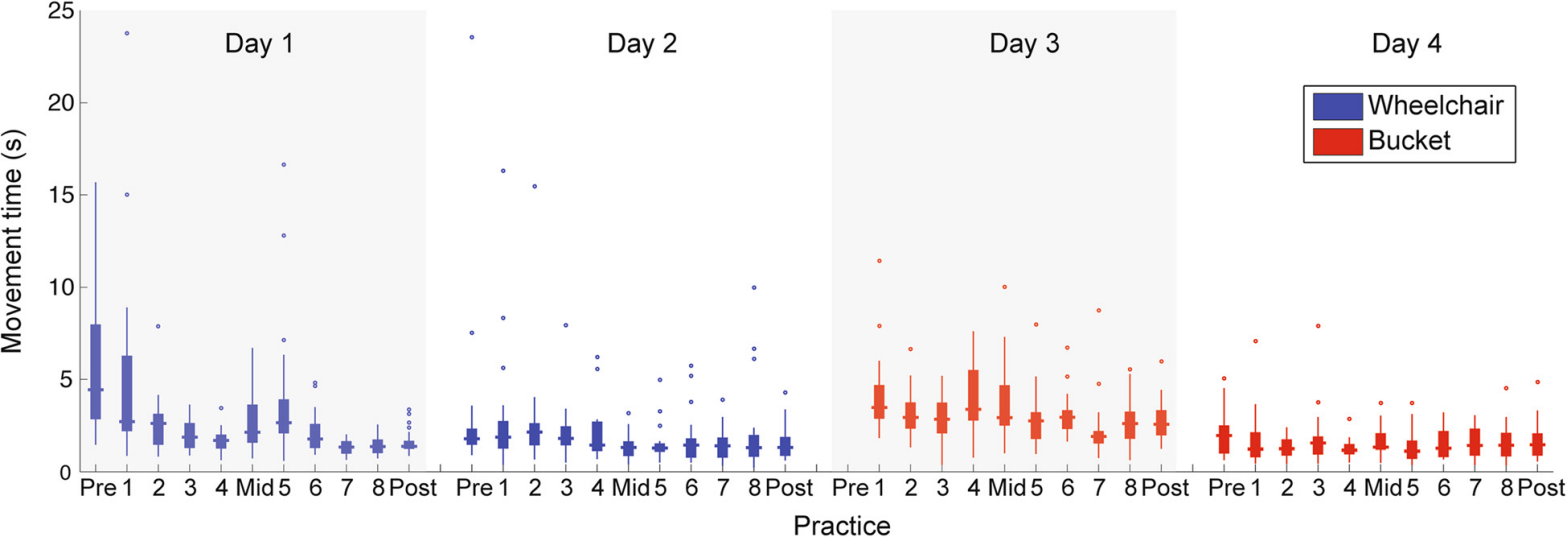
Boxplots of movement time of Z in each block in the wheelchair condition (days 1 and 2) and in the bucket condition (days 3 and 4). Each testing session was separated by 1 week. Testing sessions consisted of 24 trials whereas training sessions consisted of 20 trials each. Each day consisted of a pre-test, 4 training blocks, a mid-test, 4 more training blocks and a post-test. The pre-test of the bucket condition on day 3 is not displayed because of missing data. The Y-axis has been truncated to avoid 1 extreme outlier

P1 showed rapid decreases in movement time with practice, indicating that he was able to learn to control the interface quickly (Fig. 2). Because mean movement times are sensitive to the presence of outliers, we removed outliers using a detection rule using the interquartile range - i.e. an observation is an outlier if it falls outside Q1-1.5 IQR or Q3 + 1.5 IQR, where Q1, Q3 are the first and third quartiles [27]. The decrease in movement time with practice was statistically significant (Z = -5.217, *p* < .001). In addition to almost reaching a plateau in task performance within a single day, P1 also showed significant retention of performance even after 1 week (Fig. 3). Movement times in the pre-test on Day 2 were significantly shorter than the pre-test on Day 1 (Z = -3.867, *p* < .001). Finally, when examining robustness, we found that movement times in the bucket condition (mid-test Day 3) were significantly longer than corresponding block in the wheelchair condition (mid-test Day 1) (Z = -2.557, p = .011). However the magnitude of the difference in median movement time was quite small (2.12 s in the wheelchair vs. 2.91 s in the bucket).

### Normalized path length

**Fig. 4:**
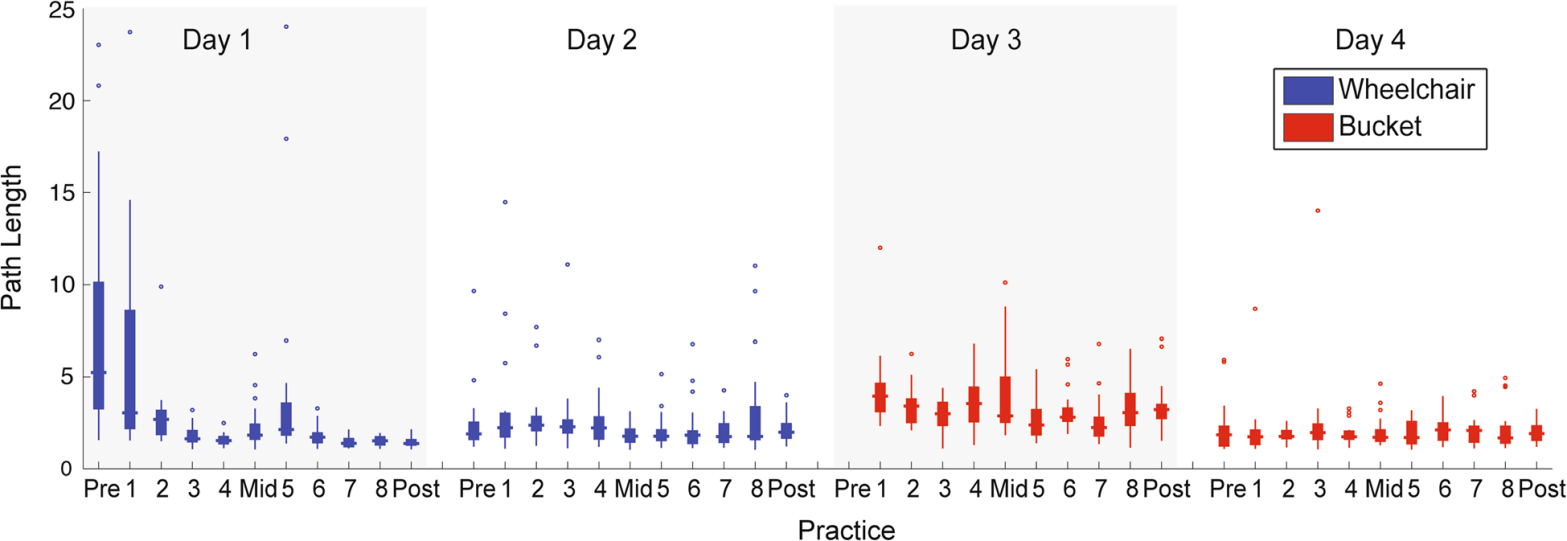
Boxplots of path length of Z in each block in the wheelchair condition (days 1 and 2) and in the bucket condition (days 3 and 4). Each testing session was separated by 1 week. Testing sessions consisted of 24 trials whereas training sessions consisted of 20 trials each. Each day consisted of a pre-test, 4 training blocks, a mid-test, 4 more training blocks and a post-test. The pre-test of the bucket condition on day 3 is not displayed because of missing data. The Y-axis has been truncated to avoid 7 extreme outliers

Normalized path length showed almost identical trends to movement time (Fig. 4). Movement paths were significantly straighter with practice on Day 1 (Z = -5.774, *p* < .001), and were retained 1 week later (Z = -4.310, *p* < .001). Also, paths in the bucket condition (mid-test Day 3) were significantly longer than the corresponding block in the wheelchair condition (mid-test Day 1) (Z = -3.629, *p* < .001). However, once again the magnitude of this difference was quite small (1.82 in the wheelchair vs. 2.87 in the bucket).

## Discussion

We examined the learning of a cursor control task in a child P1, who was born without upper and lower limbs. P1 was able to control the position of the cursor with his upper-body movements using a body-machine interface based on IMUs. Results showed that P1 learned the task quite rapidly, decreasing movement time, and reaching a performance plateau almost within a single session of practice. These results corroborate and extend previous research showing that the interface was effective in adults with high-level spinal cord injuries with tetraplegia [5]. In addition, we also examined the robustness of the interface to changes in trunk orientation and found that P1 was able to successfully adapt to different changes in trunk posture without the need for interface recalibration, although movement times were slightly longer when the trunk posture was changed. The robustness of the map arises from two important design features – (i) the map is linear, and therefore allows the user to quickly adapt to slight changes in body posture, and (ii) the map allows for redundancy, which means that the user can adopt multiple body postures to get the cursor into any particular target. Finally, although we did not explicitly manipulate variability in positioning the IMUs, the consistent day-to-day performance demonstrates that the interface is also robust to small changes in the location of the IMUs relative to the body.

Interestingly, there was evidence of superior performance in P1 relative to age-matched typically developing children. Several factors are likely to contribute to this result: first, P1 may have better control of his upper body because of cortical reorganization due to his congenital absence of limbs, expanding cortical maps involved in trunk control into areas normally occupied by upper and lower limbs; such large-scale changes of cortical representations have been previously demonstrated [3, 15]. At the same time, while there has been strong evidence of reorganization in amputees, there is still debate about the degree to which cortical reorganization occurs in people with congenital limb deficiency [6, 11, 20]. Second, P1 may have engaged in more effective exploration by focusing on trunk movements whereas typically developing children also engage in exploration of movements of unrelated degrees of freedom such as the arms and hands. Finally, P1’s extensive prior experience controlling a wheelchair joystick with his upper body could have also resulted in positive transfer to this task. Further experiments are required to determine the relative contribution of these different factors.

There are at least two potential areas for improvement to the interface. First, with respect to constructing the map, although PCA provides a principled basis to extract the space with maximum variance, there are limitations with this technique, such as (i) PCA being linear and therefore potentially not being able to capture inherent non-linearities in the movements, and (ii) the orthogonality constraint of PCA resulting in cases where movements may be non-intuitive or difficult to do. Some of these issues can be alleviated by (i) ensuring that the IMUs are able to capture a sufficiently rich set of behaviors (i.e., that there is sufficient redundancy in the system) and (ii) by customizing the map in cases of non-intuitive or difficult control (for example, by applying a scaling or rotation to the map). A second area for improvement is the robustness of learning – we found small but statistically significant differences in movement times between bucket and wheelchair conditions, suggesting that once the participant learned to control the interface in one posture, learning to adjust to a slightly different posture was difficult. One reason for this was that there was decreased postural stability in the bucket prosthesis condition, which may have limited some movements that P1 could make. However, one way to alleviate this issue of robustness is by using a random or intermittent practice schedule [22, 25], where participants switch back and forth between different postures when learning to control the cursor. This is in contrast to the blocked practice schedule used in the current study, where P1 first learned to control the cursor in one posture, followed by the other posture.

## Conclusions

The body-machine interface developed here is especially appealing for children because it (i) is non-invasive and simple to use (requiring the user only to wear 4 IMU sensors), (ii) has a simple automated calibration procedure (requiring only about 1 min of data the first time it is worn, and does not need to be constantly recalibrated), (iii) is easy to learn (within the course of an hour), and the acquired sensorimotor map is well-retained even after a period of one week, and (iv) is reasonably robust to changes in trunk orientation, indicating that the interface is feasible for everyday use, enabling the individual to assume a variety of postures without compromising the functionality of the interface. Moreover, unlike head-, gaze-, and mouth-controlled devices, the interface does not interfere with communication or visual attention, thereby facilitating the social interaction of the child with family and peers. The ability to maintain social interaction has been identified as a critical factor for continued use of an assistive device in children [13]. Finally, the interface can also be dynamically adapted to account for changes due to physical growth and movement repertoire by simply recalibrating the map. An important future extension of this work is to progress from 2-D control of a screen cursor to the control of the end-effector of a robotic arm moving in 3-D space.
